# Chromatibody, a novel non-invasive molecular tool to explore and manipulate chromatin in living cells

**DOI:** 10.1242/jcs.183103

**Published:** 2016-07-01

**Authors:** Denis Jullien, Julien Vignard, Yoann Fedor, Nicolas Béry, Aurélien Olichon, Michèle Crozatier, Monique Erard, Hervé Cassard, Bernard Ducommun, Bernard Salles, Gladys Mirey

**Affiliations:** 1 Toxalim, Université de Toulouse, INRA, Université de Toulouse 3 Paul Sabatier, 31027 Toulouse, France; 2 ITAV, Université de Toulouse, CNRS, UPS, 31106Toulouse, France; 3 CRCT-UMR1037, Université de Toulouse, INSERM, 31037 Toulouse, France; 4 Centre de Biologie du Développement UMR5547, 31062 Toulouse, France; 5 IPBS-UMR5089, Université de Toulouse, CNRS, 31077 Toulouse, France; 6 IHAP, Université de Toulouse, INRA, ENVT, 31076 Toulouse, France; 7 CHU de Toulouse, 31106 Toulouse, France

**Keywords:** Chromatin, Real-time imaging, Single-domain antibody, Chromatin function, Epigenetic

## Abstract

Chromatin function is involved in many cellular processes, its visualization or modification being essential in many developmental or cellular studies. Here, we present the characterization of chromatibody, a chromatin-binding single-domain, and explore its use in living cells. This non-intercalating tool specifically binds the heterodimer of H2A–H2B histones and displays a versatile reactivity, specifically labeling chromatin from yeast to mammals. We show that this genetically encoded probe, when fused to fluorescent proteins, allows non-invasive real-time chromatin imaging. Chromatibody is a dynamic chromatin probe that can be modulated. Finally, chromatibody is an efficient tool to target an enzymatic activity to the nucleosome, such as the DNA damage-dependent H2A ubiquitylation, which can modify this epigenetic mark at the scale of the genome and result in DNA damage signaling and repair defects. Taken together, these results identify chromatibody as a universal non-invasive tool for either *in vivo* chromatin imaging or to manipulate the chromatin landscape.

## Abstract (precis)

**Summary:** Chromatibody is a chromatin-binding single-domain antibody, derived from llama nanobodies, that can be used as a novel non-invasive molecular tool to explore and manipulate chromatin in living cells.

## INTRODUCTION

Histones are the basic structural components of chromatin. Eukaryotic DNA is wrapped around histone octamers, containing two copies of each of the core histones (H2A, H2B, H3 and H4) (Luger et al., 1997). Histones are abundant, small basic proteins that can be covalently modified at their N- or C-terminal tails, as well as on their globular domains (Bannister and Kouzarides, 2011). Histone post-translational modifications modulate their interaction with both DNA and effector proteins, therefore influencing chromatin structure and function (Bhaumik et al., 2007; Rothbart and Strahl, 2014). Chromatin function and dynamics are involved in many cellular processes, including gene expression regulation, DNA repair or meiosis. Visualizing chromatin in living cells and model organisms is a key step in many studies (Keller, 2013). Thus, the expression of a fluorescently-tagged histone has been a cutting-edge technique to visualize chromatin in cultured cells (Kanda et al., 1998), as well as in model organisms (Clarkson and Saint, 1999; Pauls et al., 2001) and still remains a common approach to study chromosome dynamics, mitosis or even apoptosis during developmental processes. Fluorescent-tagged histones that incorporate into nucleosomes have also been used to study their exchange dynamics within chromatin fiber (Kimura and Cook, 2001; Bonisch et al., 2012).

The development of core-histone-binding molecules that interact with the nucleosomes in living cells constitutes a major issue in biological and medical research, as it could be used to deliver specific activities and modulate chromatin functions (Helin and Dhanak, 2013). Currently, recruiting an activity of interest to nucleosomes mainly relies on expressing fusion proteins, in which the targeting unit is an endogenous chromatin component, typically a core histone. Until recently, this was achieved by overexpression, but genome-editing tools (Boch et al., 2009; Cong et al., 2013) are about to change the situation (Dean and Palmer, 2014; Ratz et al., 2015). However, tagging endogenous proteins still raises concern about the invasiveness of this approach. Because histones, in their native form, are involved in essential and highly regulated functions, it calls for the development of alternative, fully exogenous core-histone-binding molecules and highlights the need for alternative probes to study chromatin.

Single-domain antibodies (sdAbs) or nanobodies have been shown to be an excellent tool to generate small monomeric binding domains against an antigen of interest (Muyldermans, 2013; De Meyer et al., 2014). sdAbs derive from the monomeric variable antigen-binding region (VHH) of heavy-chain-only antibodies naturally produced by the immune system of camelids (Hamers-Casterman et al., 1993), from some selective human VH domains (Tanaka et al., 2003), or even IgNar from sharks (Dooley et al., 2003). sdAbs directed against an antigen of interest are usually isolated from immune, natural or semi-synthetic VHH libraries, using display methods (Arbabi Ghahroudi et al., 1997; Doshi et al., 2014; Fridy et al., 2014). Characterized by a convex paratope that allow them to bind epitopes localized inside molecules clefts or cavities (De Genst et al., 2006), some VHHs have also been reported to be sensitive to the conformational state of their target antigen (Tanaka et al., 2007; Kirchhofer et al., 2010; Irannejad et al., 2013). Most interestingly, because of their folding properties and high stability, sdAbs usually remain functional and are still able to bind their antigen when expressed in living cells. This unique characteristic has been exploited to trace and image intracellular antigens, in living cells, by expressing fluorescently-tagged sdAbs (Rothbauer et al., 2006), and to follow DNA replication (Burgess et al., 2012), apoptosis (Zolghadr et al., 2012) and HIV virion assembly (Helma et al., 2012). Remarkably, these approaches unveiled a new imaging strategy, avoiding the modification of the endogenous proteins with a fluorescent tag. In addition, the ability of the sdAb to target an enzymatic activity has been demonstrated: a GFP-binding VHH, fused to an E3 ubiquitin-ligase, induced the proteasome-dependent degradation of the GFP-tagged proteins (Caussinus et al., 2012). Thus, sdAbs appear to be unique recombinant binding molecules for cell biology studies (Helma et al., 2015), allowing the delivery of a fluorophore or an enzymatic activity to visualize or manipulate specific intracellular components.

Here, we establish a generic tool to explore chromatin, without overexpressing or interfering with endogenous nucleosomal content. We developed sdAbs that have the unique ability to bind the H2A-H2B heterodimer *in vitro* and *in vivo*. These sdAbs specifically label chromatin from yeast to human, when used in immunostaining, and were therefore named chromatibody. Furthermore, expression of GFP-tagged chromatibody in human cells or in a transgenic animal model allows *in vivo* high-resolution real-time chromatin imaging and visualization of chromosome dynamics. Finally, we show that chromatibody can be used to target the E3 ubiquitin ligase RNF8 to the nucleosome to globally modify the epigenetic marks at the genomic level. We report a global DNA damage-dependent H2A ubiquitylation, leading to DNA damage signaling and repair alteration. Taken together, these results establish chromatibody as a universal non-invasive recombinant tool for chromatin imaging, and show that it can be used to modify epigenetic marks at the whole-genome scale.

## RESULTS

### Chromatibody binds the H2A–H2B histone heterodimer

**Fig. 1.**
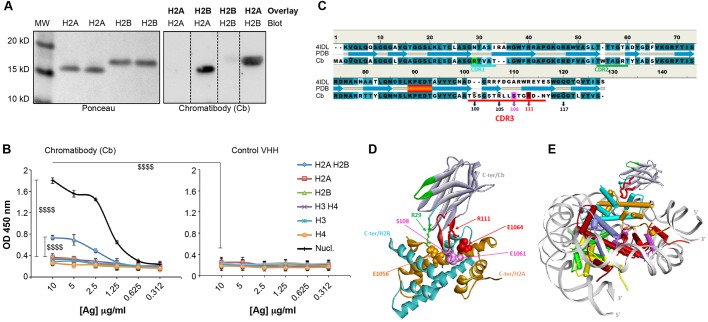
**Specificity of the chromatibody binding.** (A) Purified histones H2A or H2B were transferred after SDS-PAGE. The left panel shows the H2A and H2B histones upon Ponceau staining. The overlay was performed with the purified H2A or H2B histones, followed by the incubation with chromatibody revealed by anti-HA and the HRP-conjugated secondary antibody (right panel). (B) The chromatibody (Cb) binding specificity to purified core histones (H2A, H2B, H3 or H4), the H2A–H2B dimer, the H3–H4 tetramer or nucleosomes (Nucl.) was assessed by ELISA assays. A control VHH was used as a negative control (right panel). Histone concentrations coated on the plates are indicated (antigen [Ag] concentrations are in µg/ml). Three independent experiments were performed (*n*=3), each point as triplicates. Results are mean±s.e.m. $$$$, *P*<0.0001 [for the statistical significance between the chromatibody binding to nucleosomes, H2A–H2B or the other antigens indicated (Ag concentration of 10 µg/ml), and compared to control VHH]. (C–E) Modeling of the interaction between chromatibody and the H2A–H2B dimer. (C) Secondary-structure-guided sequence alignment between the anti-cholera toxin VHH structural template (PDB code: 4IDL) and the chromatibody chromatibody. The three complementary determining regions (CDRs) are delineated under the alignment (turquoise, green and red, respectively) and are similarly color-coded in the optimized view of the 3D models. (D) Prediction of the complex between chromatibody and the H2A–H2B dimer. Three acidic residues from the H2A second helix (displayed in CPK mode), E1056 (brown), E1061 (purple) and E1064 (red), form hydrogen-bonds with chromatibody R29 (green), S108 (pink) and R111 (red), respectively. Hydrogen bonds between chromatibody and H2B (marked by black arrowheads, Fig. 1C) strengthen the interaction. (E) Modeled structure resulting from docking the chromatibody CDR3 hairpin into the H2A–H2B acidic cavity at the surface of the nucleosome core.

In an attempt to select sdAbs allowing DNA double-strand break detection, we used a phage-display selection against phosphorylated H2AX (γH2AX) (Fig. S1A). This strategy led us to identify an sdAb that marked chromatin (Fig. S1B) and recognized a protein of ∼15 kDa that is not γH2AX (Fig. S2A,B). Moreover, immunodetection assays in H2AX^−/−^ MEFs (Celeste et al., 2002) showed that this sdAb was not specifically directed against H2AX (Fig. S2C,D). According to the electrophoretic migration of the target and the selection strategy, we hypothesized that this sdAb might recognize H2A or H2B histones. To test this, we performed an overlay experiment (also known as far-western) and showed that the sdAb only recognized the H2A–H2B dimer (Fig. 1A). In addition, a standard western blot analysis, with single core histones or with the H2A–H2B heterodimer, was performed and confirmed these results (Fig. S2E). Finally, to determine its binding specificity, the sdAb was used to probe immobilized individual core histones, H2A–H2B dimers, H3–H4 tetramers and mononucleosomes in an indirect ELISA assay (Fig. 1B). In contrast to a conventional H2B antibody that either binds H2B or the H2A–H2B heterodimer (Fig. S2F), the selected sdAb only interacted with H2A–H2B and mononucleosomes. Therefore, we called it chromatibody.

In order to gain insights into the mode of interaction of chromatibody with the H2A–H2B dimer and nucleosomes, we performed modeling studies (Fig. 1C–E). The three-dimensional structure of chromatibody was first built based on its sequence and secondary structure similarity with the crystallographic structure of the llama anti-cholera toxin VHH domain (Legler et al., 2013) (Fig. 1C). A distinctive feature in both cases was the β-hairpin structure of the third complementary determining region (CDR3) loops. Interestingly, the chromatibody hairpin encompasses the β-turn-containing motif RL**LSTG** (residues R105 to G110), which is structurally reminiscent of the chromatin-binding motif (CBM) MX**LRSG** identified in Kaposi's sarcoma herpes virus latency-associated nuclear antigen (LANA) and in interleukin-33 (Il-33) (Barbera et al., 2006; Roussel et al., 2008). We then explored, by performing molecular docking, whether the CBM-like motif present in chromatibody could mediate its interaction with chromatin. Using the crystallographic structure of the complex between the nucleosome core and LANA CBM (Barbera et al., 2006) as a framework to position chromatibody relatively to the H2A–H2B dimer, we obtained a stable structure, without steric clash, of the complex between chromatibody and the H2A–H2B dimer, either free (Fig. 1D) or in the core particle (Fig. 1E). A crucial pair of acidic residues from the H2A second helix (E1056 and E1064) directs chromatibody binding to the H2A–H2B dimer through both electrostatic and hydrogen-bond interactions, involving R29 and R111, respectively. Most importantly, the tight β-turn LSTG at the tip of the chromatibody CDR3 loop is tethered to the H2A second helix through a hydrogen-bond between chromatibody S108 and H2A E1061. Finally, hydrogen bonds between chromatibody S100, R105, R111 and Q117 (marked by black arrowheads in Fig. 1C) and the H2B backbone strengthen the interaction.

### Chromatibody is a universal tool to label chromatin

**Fig. 2.**
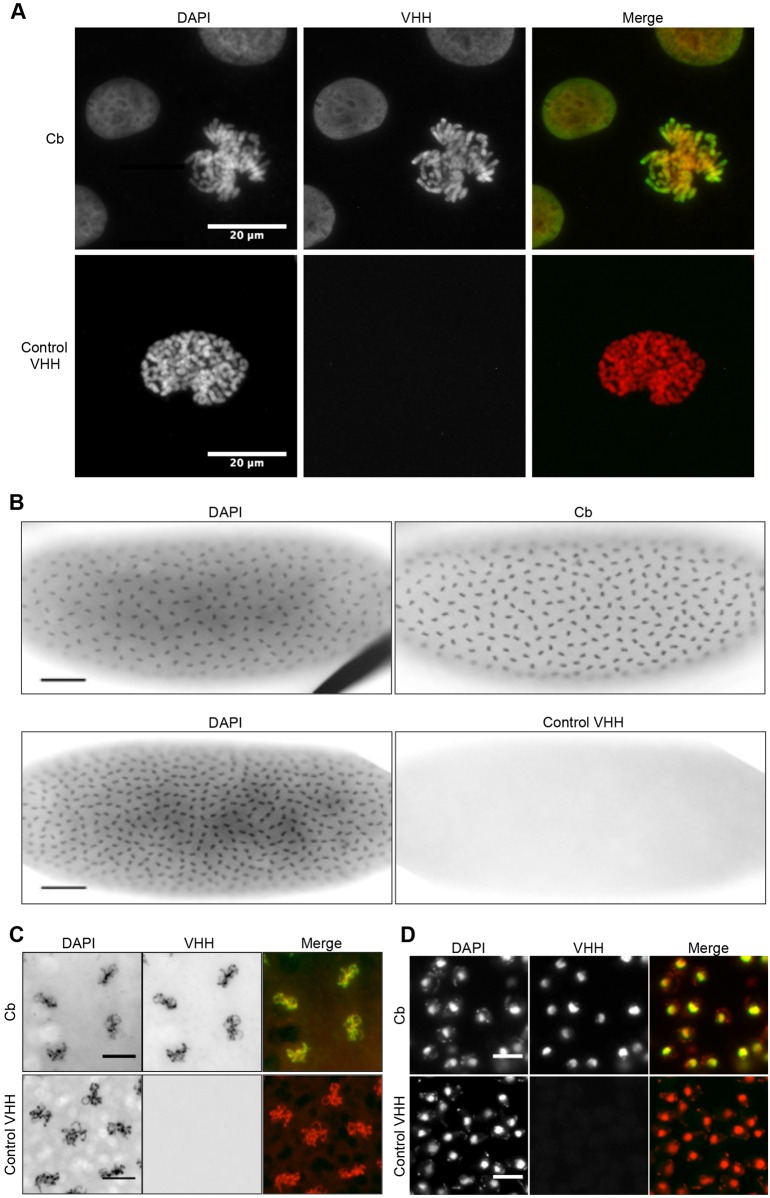
**Chromatin staining in different model systems.** Chromatibody (Cb) allows the immunostaining of chromatin in a wide interspecies system. In the merged images, VHH and DAPI signals are shown in green and red, respectively. Control VHH was used as a negative control. (A) HCT116 cells were immunostained with chromatibody or control VHH, and DNA was labeled with DAPI. The right panels (merge) show both DAPI and VHH stainings. (B) Fluorescence pictures (inverted gray scale) of blastoderm *Drosophila* embryos immunostained with chromatibody or control VHH. Scale bars: 50 µm. (C) Higher magnification of the stained embryos and overlap between VHH and DAPI (merge). Scale bars: 10 µm. (D) Budding yeast cells stained with chromatibody or control VHH and DAPI. Scale bars: 5 µm.

In order to assess the possibility of using chromatibody *ex vivo*, immunofluorescence experiments on fixed and permeabilized cells of different model systems were performed. In HCT116 (human colon carcinoma) mitotic cells, only chromosomes were stained, whereas in interphasic cells, chromatibody homogenously stained the nucleus and was partially excluded from the nucleoli (Fig. 2A). This signal was similar to DAPI staining and was specific, as no staining was observed with the control VHH. We next asked whether chromatibody might recognize chromatin in model organisms that were evolutionarily distant from mammals. Immunostaining of *Drosophila melanogaster* blastoderm embryos with the chromatibody resulted in a specific chromatin staining, allowing high resolution imaging of the mitotic chromosomes (Fig. 2B,C). Chromatin-specific staining was also observed both in *Caenorhabditis elegans* (data not shown) and in the evolutionarily distant eukaryote, *Saccharomyces cerevisiae* (Fig. 2D). Taken together, these data establish the universal ability of chromatibody to bind chromatin.

### Expression of GFP-tagged chromatibody allows chromatin live imaging

**Fig. 3.**
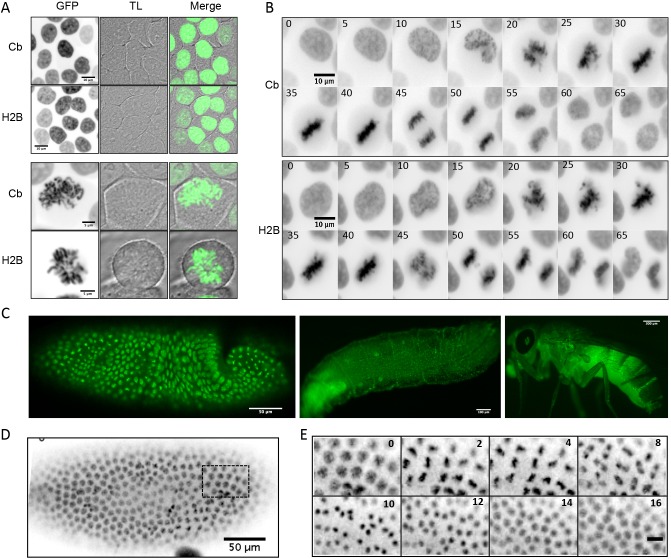
**Chromatibody–GFP fusion expressed in living cells.** (A) Confocal images of HCT116 cells expressing chromatibody (Cb)–GFP or H2B–GFP at interphase (upper panels, scale bars: 10 µm) or mitosis (lower panels, scale bars: 5 µm). Left, middle and right panels show the GFP fluorescence (inverted gray scale), transmitted light (TL) and merged signals, respectively. (B) Time-lapse fluorescence imaging (inverted gray scale) of HCT116 cells stably expressing the chromatibody–GFP or H2B–GFP. The time sequence is indicated in minutes. (C) Confocal imaging of a *Drosophila* blastoderm (scale bar: 50 µm), living larvae (scale bar: 100 µm) and adult (scale bar: 300 µm) expressing the chromatibody–GFP under the control of the tubulin promoter. (D) Fluorescence image (inverted gray scale) of a living *Drosophila* embryo expressing chromatibody–GFP. (E) Higher magnification and time-lapse fluorescence microscopy of the embryo shown in D. Scale bar: 10 µm. The time sequence is indicated in minutes.

One of the most remarkable properties of sdAbs is their propensity to be used as intrabodies, that is, to bind intracellular antigens *in vivo* (Rothbauer et al., 2006). To determine whether chromatibody is able to bind chromatin when used as an intrabody, we constructed C-terminally GFP-tagged versions and stably expressed them in different model systems. We anticipated that because the chromatibody–GFP fusion is relatively small (around 42 kDa) it should freely diffuse through the nuclear pore complexes. Thus, no nuclear localization sequence was introduced in the construct. Using fluorescence confocal microscopy in living HCT116 cells, we analyzed chromatin labeling when chromatibody–GFP, or histone H2B–GFP fusion (Kanda et al., 1998), was expressed. We observed that both interphase chromatin and mitotic chromosomes labeling were similar (Fig. 3A). We then performed time-lapse fluorescence microscopy to evaluate the ability of chromatibody–GFP to allow non-invasive imaging of the chromatin in living cells. This revealed that the chromatibody–GFP probe remained specifically associated with chromatin throughout cell division, as we never observed any cytoplasmic labeling (Fig. 3B; Movie 1). The chromatibody–GFP expression did not perturb progression through mitosis, and the mitotic timing was comparable to the H2B–GFP fusion (Fig. 3B). The growth curves established with HT1080 cells stably expressing chromatibody–GFP are similar to those of the parental HT1080 cells (Fig. S3A). Cells stably expressing the fluorescent chromatibody could be passaged for up to 2 months without substantial cell death or loss of transgenic expression (data not shown). ­Finally, stable expression of chromatibody–GFP does not alter the cell cycle kinetics of synchronized cells (Fig. S3B). Taken together, these data strongly suggest that the expression of the fluorescent chromatibody does not affect either cell cycle progression or cell viability.

To explore the potential for using the non-invasive chromatibody during development of a multicellular organism, we generated transgenic *Drosophila* that expressed GFP-tagged chromatibody. Time-lapse imaging of developing blastoderm embryos expressing chromatibody–GFP was performed. Chromatibody associated with chromatin and allowed its visualization throughout development, from embryo to larval and adult stages (Fig. 3C). Embryos expressing chromatibody–GFP developed normally, giving rise to fluorescent larva, pupae and adults in which nuclei, even those of internal structures (and especially those of the salivary glands where endoreplication takes place), are clearly labeled (Fig. S3C). The specificity of the labeling allowed a high resolution tracking in space and time of the chromatin dynamics, including during cell divisions (Fig. 3D,E) and movement of nuclei that occur at gastrulation (Fig. S3D; Movie 2). Taken together, these data support that the chromatibody expression and its association to chromatin does not interfere with cell viability, cell division or the developmental program in *Drosophila*.

### Chromatibody is a dynamic chromatin probe that can be modulated

**Fig. 4.**
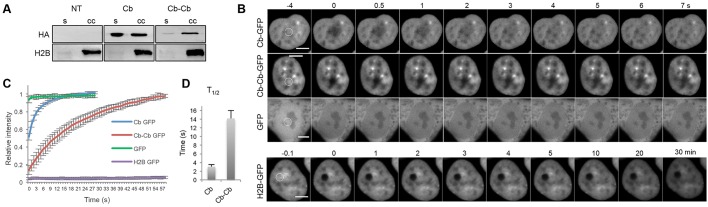
**Chromatibody dynamic properties.** (A) Parental HCT116 cells (NT, not transfected) or HCT116 cells stably expressing chromatibody–GFP (Cb) or the bivalent chromatibody–chromatibody–GFP (Cb-Cb) were subjected to biochemical fractionation. The soluble (s) and crude chromatin (cc) fractions were analyzed by western blotting with antibodies against the HA tag or H2B. (B) Images showing the fluorescence recovery after photobleaching (FRAP) kinetics in the nucleus of HCT116 cells expressing the chromatibody–GFP, chromatibody–chromatibody–GFP, GFP or H2B–GFP. The photobleached areas are indicated by the dashed circle. GFP and H2B–GFP were used as highly mobile and low-mobile controls, respectively. The time sequence is indicated. Scale bars: 5 µm. (C) FRAP curves established from HCT116 cells expressing the GFP constructs mentioned on the right. Mean±s.d. intensities in the photobleached area relative to pre-bleaching intensities are plotted as a function of time after bleaching (*n*=9). (D) Histogram showing the mean±s.d. recovery half time (*t*_1/2_) calculated for chromatibody–GFP and bivalent chromatibody–chromatibody–GFP from the FRAP experiments shown in C.

We explored the dynamic properties of chromatibody and bivalent chromatibody, in order to introduce avidity and thus engineer binding molecules with increased functional affinity (Olichon and Surrey, 2007; Hultberg et al., 2011; Cardoso et al., 2014). The bivalent construct was composed of two chromatibody copies, separated by a llama IgG2c hinge flexible linker, and tagged with GFP. Despite the size increase of the construct, the bivalent chromatibody still specifically localized in the nucleus of interphasic cells, without any NLS, and labeled chromosomes in mitotic cells (Movie 3). Cells expressing chromatibody–GFP or the bivalent chromatibody–chromatibody–GFP were subjected to biochemical fractionation of soluble cytosolic and nucleoplasmic fractions from crude chromatin. Western blot analyses of these fractions revealed that, whereas H2B was restricted to the insoluble fraction, chromatibody was only partially associated with the chromatin fraction, with a substantial amount of chromatibody found in the soluble material. Instead, the bivalent chromatibody, which might be expected to display an enhanced avidity for its target, is almost totally associated with the chromatin fraction (Fig. 4A). To further characterize the association of chromatibody to chromatin, we performed fluorescence recovery after photobleaching (FRAP) experiments (Fig. 4B). The experimental conditions used here permitted observation of the low mobility of H2B–GFP (Fig. 4B, lower panel), as described previously (Kimura and Cook, 2001). The chromatibody exhibited a fast recovery (Fig. 4B) that was, however, slower than that of GFP alone (Misteli et al., 2000; Phair et al., 2004; Bonisch et al., 2012). The rapid and complete recovery of the intensity in the bleached zone shows the absence of an immobile or slow-moving fraction of the chromatibody (Fig. 4C). The bivalent chromatibody presented a lower mobility exchange (Fig. 4B), with a slower recovery dynamics (Fig. 4C). The apparent free fraction (∼14%) of the bivalent chromatibody is smaller than that of the single chromatibody, consistently with its tight association to chromatin (Fig. 4A). These differences can also be observed by comparing Movie 1 (cells expressing chromatibody–GFP) and Movie 3 (cells expressing bivalent chromatibody–GFP, where chromatin condensation in interphasic cells is observable). Mono-exponential fitting of these FRAP data allowed the calculation of the half-time recovery value of 2.9 s for the chromatibody and 14 s for the bivalent chromatibody (Fig. 4D). Our experimental set-up did not allow us to estimate the *t*_1/2_ for GFP or H2B–GFP as these were, respectively, too fast and too low to be calculated. Thus, these data indicate that the chromatibody interaction with chromatin is highly dynamic, being far less tightly associated to chromatin than H2B–GFP, suggesting that they are labile probes for chromatin studies. To summarize, these results show that chromatibody is a dynamic chromatin probe that can be modulated for its binding properties.

### Chromatibody allows modifying the cell response to DNA damage

**Fig. 5.**
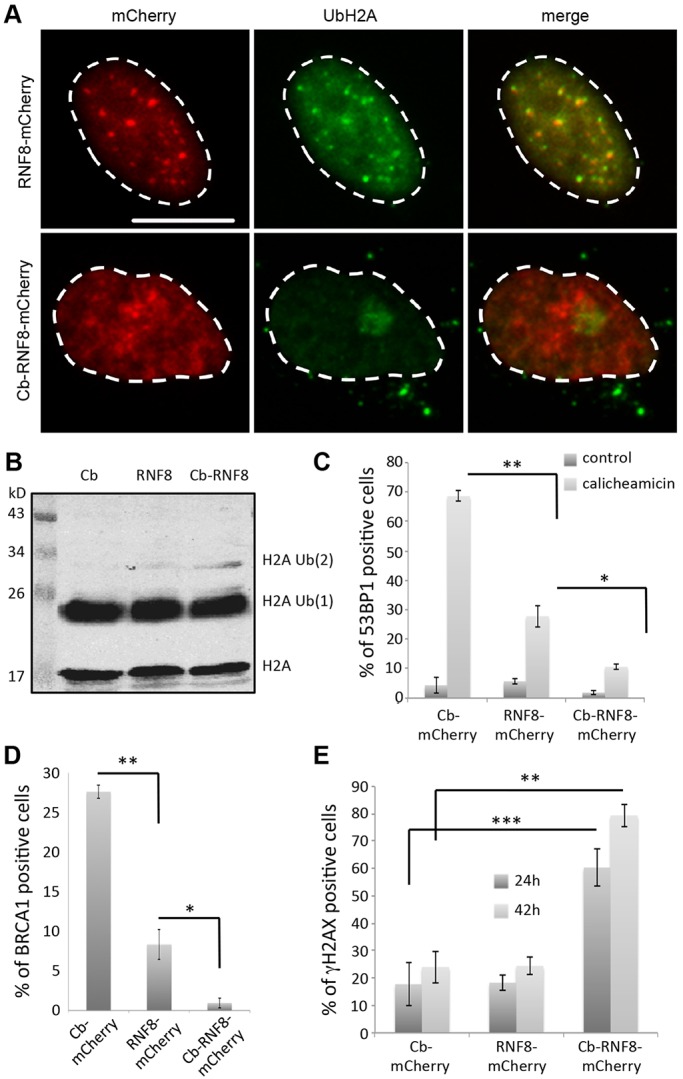
**Chromatibody-driven DDR alteration.** (A) DNA-damage-dependent RNF8 and UbH2A foci. HeLa cells were transfected with the indicated forms of RNF8 24 h prior treatment with calicheamicin (10 pM) and immunostained for ubiquitylated H2A (UbH2A). Cb, chromatibody. Scale bar: 20 µm. (B) RNF8-dependent H2A di-ubiquitylation. HT1080 cells were transfected with the indicated constructs for 24 h. Whole-cell extracts were analyzed with an anti-ubiquitinylated H2A antibody (UbH2A). (C) 53BP1 recruitment to DSBs. HT1080 cells were transfected with the indicated forms of RNF8 for 24 h, treated (10 pM calicheamicin) and immunostained for 53BP1. (D) BRCA1 foci formation. HT1080 cells were transfected with the indicated forms of RNF8 for 24 h and fixed before being immunostained for BRCA1. (E) Accumulation of spontaneous unrepaired DSBs. HT1080 cells were transfected with the indicated constructs for 24 or 42 h and immunostained for γH2AX without any genotoxic treatment. The graphs are mean±s.d. from three independent experiments (*n*=3) in which at least 100 cells for each category were scored. *P<0.05, **P<0.01, ***P<0.001 (Student's *t*-test).

We next investigated the possibility of using chromatibody to target an enzymatic activity to chromatin. As a proof of concept, we fused chromatibody to RNF8, an E3 ubiquitin ligase that is targeted to DNA double-strand break (DSB) sites to induce H1 ubiquitylation and initiate RNF168-mediated H2A and H2AX polyubiquitylation, an essential step for DSB signaling and repair (Mailand et al., 2007; Stewart et al., 2009; Mattiroli et al., 2012; Thorslund et al., 2015). Upon genotoxic treatment [10 pM calicheamicin, a DSB-inducing agent (Elmroth et al., 2003) or γ-irradiation], the mCherry–RNF8 fusion relocalizes as nuclear foci to DSB sites, where it colocalizes with antibodies directed either against ubiquitylated H2A (Fig. 5A) or against ubiquitin conjugates (FK2 antibody) (Fig. S4A). However, chromatibody N-terminally fused to RNF8 no longer accumulated at DSB sites, preventing the localized H2A ubiquitylation. This defect was not due to the impaired capacity to ubiquitylate H2A, as the global level of ubiquitylated H2A was similar between cells expressing the chromatibody, RNF8 or the chromatibody–RNF8 construct (Fig. 5B), with the chromatibody–RNF8 expression even enhancing the level of di-ubiquitylated H2A. These results show that chromatibody targets RNF8 to the whole chromatin, therefore preventing its normal accumulation to damaged DNA sites. As a result, RNF8-dependent H2A ubiquitylation is no longer concentrated to DSBs but is slightly increased at the whole genome scale, especially H2A di-ubiquitylation.

Di-ubiquitylated H2A is the preferred form recognized by RNF168 to amplify H2A polyubiquitylation and recruit the downstream DSB repair factors 53BP1 (also known as TP53BP1) and BRCA1 (Stewart et al., 2009). Therefore, misregulated H2A ubiquitylation might lead to defective 53BP1 and BRCA1 recruitment to DSBs. Calicheamicin-treated cells failed to properly form 53BP1 foci when overexpressing mCherry–RNF8, this defect being exacerbated when the chromatibody fusion is expressed (Fig. 5C; Fig. S4B). In the same way, the formation of BRCA1 foci that occurs during unperturbed S-phase (Scully et al., 1997) was impaired by RNF8 overexpression, and this defect was strongly increased when RNF8 was targeted to chromatin by the chromatibody (Fig. 5D; Fig. S4C). In conclusion, altered recruitment of RNF8 due to its fusion with chromatibody prevents proper 53BP1 and BRCA1 foci formation.

Finally, we investigated the capacity of chromatibody–mCherry–RNF8-expressing cells to repair DSBs. Surprisingly, even in the absence of exogenous genotoxic insult, cells expressing the chromatibody–RNF8 fusion form γH2AX foci in a time-dependent manner compared to cells expressing either the chromatibody or RNF8 (Fig. 5E). These data illustrate the major DSB repair deficiency is linked to the expression of the chromatibody–RNF8 fusion. In summary, our results clearly demonstrate that chromatibody mediates inappropriate targeting of RNF8 to chromatin resulting in the dramatic corruption of DSB signaling and repair.

## DISCUSSION

Histones are fundamental components of chromatin that are covalently modified at their tails or at their globular domains (Bannister and Kouzarides, 2011). These post-translational modifications modulate the interaction of histones with DNA and effector proteins, therefore impacting upon chromatin structure and function (Bhaumik et al., 2007; Rothbart and Strahl, 2014). Here, we aimed to establish a generic tool to explore chromatin dynamics and function, without overexpressing or interfering with the endogenous nucleosomal content. We therefore chose to develop sdAbs, and characterize in detail one sdAb, which specifically bound the H2A–H2B heterodimer and we called chromatibody.

We first showed that chromatibody recognizes the H2A–H2B heterodimer both *in vitro* and *ex vivo*. Genetically encoded chromatibody–GFP, when expressed in living cells, could be used as fluorescent probe to visualize chromatin from yeast to mammals. This broad species cross-reactivity to chromatin might be due to the fact that histones are the most evolutionary conserved components of the chromatin (Malik and Henikoff, 2003).

Second, we illustrated the non-invasiveness of chromatibody, by stably expressing chromatibody–GFP in human cells and in *Drosophila*. Chromatibody is a fully exogenous chromatin-binding domain; this means that the fluorescent labeling of chromatin is not associated with the genetic manipulation of chromatin core components, the replacement of an endogenous protein or the presence of an intercalating agent. As sdAbs have been shown to often recognize a cryptic epitope, through their convex shape (Nguyen et al., 2000; Stijlemans et al., 2004; De Genst et al., 2006), and because conformational reasons might explain the specific chromatibody binding to the H2A–H2B dimer, we modeled the complex. We found that the long CDR3 hairpin of the chromatibody fitted to the acidic patch of the H2A–H2B heterodimer, leaving the core histones tails free. Thus, we predict that chromatibody might not interfere with post-translational modifications of histones, among them H2A and H2B, which regulate chromatin functions and might be key for the non-invasive properties of chromatibody. Histone tails are particularly important, with the histone H2A C-terminal tail regulating the chromatin dynamics (Vogler et al., 2010), the H2AZ variant C-terminus regulating its function and association to nucleosomes (Wratting et al., 2012), and the H2B tail playing an important role in transcriptional initiation and elongation through its ubiquitylation (Lee et al., 2007; Kim et al., 2009). Ubiquitylation is a highly dynamic mark, regulating gene activity and silencing, but numerous post-translational modifications occur on histones (acetylation, phosphorylation, sumoylation, etc.), with some of these modifications depending on others (Lee et al., 2007; Kim et al., 2009). Finally, the 3D structure prediction of the chromatibody–nucleosome complex showed that chromatibody is a non-intercalating probe and does not interact with the minor groove of DNA, but with the H2A–H2B acidic cavity. This is a crucial point as it has been shown that intercalating agents or molecules that bind the DNA minor groove can modify the localization and the mobility properties of DNA-binding proteins (Mari et al., 2010) or cause supercoiling (Störl et al., 1993), impacting on cellular functions (Koster et al., 2010). Taken together, these data underline the non-interfering potential of the chromatibody to study chromatin dynamics without impeding DNA repair, chromatin-associated functions (e.g. replication, transcription) or epigenetic processes.

Devoid of nuclear localization signal, chromatibody spontaneously and efficiently accumulates in the nucleus whereas a control sdAb–GFP fusion homogenously localizes both in the cytoplasm and the nucleus. Thus, the chromatibody probe freely diffuses through the nuclear pores and accumulates on chromatin. However, an alternative mechanism, where chromatibody is imported into the nucleus through its binding to the H2A–H2B dimer, associated with its chaperone, cannot be fully discounted. FRAP experiments showed that there was a constant dynamic exchange between the chromatin-bound pool and the freely diffusing chromatibody nucleoplasmic fraction. This could be due to either the binding affinity to the nucleosomes in living cells being low, or because the chromatibody has a strong affinity but a weak avidity, allowing a fast ‘on and off’ exchange, as observed previously for some sdAbs (Rothbauer et al., 2006). As fluorescent chromatibody is highly mobile, it allows time-lapse imaging of chromatin in living cells over a long timeframe without photobleaching. By assembling bivalent chromatibody, we generated sensors with about a five-times lower mobility exchange compared to monovalent chromatibody. Therefore, properties of chromatibody can be modulated to produce probes with different characteristics.

Beyond targeting fluorescent proteins to chromatin for live imaging, we have explored the possibility to use chromatibody to deliver enzymatic activity and modify core histones *in vivo*. We developed a fusion between chromatibody and the E3 ubiquitin ligase RNF8. RNF8 is recruited to damaged DNA sites through interaction with phosphorylated MDC1, where it acts in concert with RNF168 to polyubiquitylate H2A, enabling the recruitment of 53BP1 and BRCA1 (Mailand et al., 2007; Stewart et al., 2009). We show that, when fused to chromatibody, RNF8 no longer accumulates at DSB sites, preventing localized H2A ubiquitylation, an epigenetic mark essential for subsequent DSB repair. However, chromatibody–RNF8 induces H2A di-ubiquitylation, suggesting that chromatibody-mediated RNF8 targeting to chromatin generates H2A ubiquitylation at undamaged sites. It has previously been shown that tethering RNF8 to a specific locus induces local H2A ubiquitylation and RNF168 accumulation, in the absence of DSBs (Luijsterburg et al., 2012). This suggests that the chromatibody–RNF8 fusion induces the uncontrolled targeting of RNF168 to the whole chromatin, and thus disperses H2A ubiquitylation away from damaged sites. Therefore, the downstream repair factors 53BP1 and BRCA1 cannot be properly recruited to DSB sites and DNA repair is blocked, resulting in DNA damage accumulation and increased γH2AX staining. Taken together, these data show that we are able to globally disturb DSB signaling and repair by targeting RNF8 to the whole chromatin by chromatibody fusion. Such a strategy could be applicable to other histone-modifying enzymes to deregulate any epigenetic mark on the entire genome.

To conclude, for the first time, we report an sdAb that recognizes a dimer of histones. Chromatibody is an alternative probe to conventional fluorescent-tagged histones, overexpression of endogenous factors or the use of an intercalating agent for chromatin labeling in living cells. Without interfering with the nucleosome structure, chromatibody allows studying or manipulating the chromatin, in a living context.

## MATERIALS AND METHODS

### VHH selection

An immune VHH library was generated through a previously published procedure (Olichon and Surrey, 2007) and screened by phage-display. Animal experimentation was conducted in 2009 as prescribed by the guidelines of the EU council for accommodation and care of animals (directive 86/609/EEC). The llama (2-year-old male *Llama glama*) was housed in the animal facilities of the National Veterinary School of Toulouse (accreditation number D 31 555 27).

Briefly, the coding sequences of the VHH repertoire of the llama, immunized for the phosphorylated γH2AX C-terminal peptide [sequence CGKKATQAS(PO3H2)QE, KLH-coupled, Eurogentec], were RT-PCR amplified from total RNAs extracted from isolated peripheral blood lymphocytes. The amplified VHH cDNA pool was ligated into the pHEN4 phagemid (Arbabi Ghahroudi et al., 1997) and electroporated into the TG1 *E. coli* strain. A total of 6.2×10^7^ colonies, resulting from the plating and the ampicillin selection of the transformed bacteria, were pooled to generate the library. Phage display selection was performed with cellular histones isolated by a high-salt extraction protocol (Shechter et al., 2007), from HT1080 cells stably expressing the H2AX histone N-terminally tagged with either a chitin-binding domain (CBD, New England Biolabs) or a tandem Strep Tag (IBA Lifesciences). The histone extracts containing CBD–H2AX and Strep2x–H2AX were incubated with chitin (New England Biolabs) and MagStrep (IBA) magnetic beads, respectively. These affinity columns were successively used in two consecutive rounds of phage display.

For VHH characterization, cell immunostaining was used. Colonies generated after the second round of phage display were randomly picked to seed a 96-well plate filled with 1 ml of 2TY medium supplemented with 0.1% glucose and 100 µg/ml ampicillin. The periplasmic production of the VHH clones, C-terminally tagged with HA, was induced by addition of 0.5 mM IPTG. After centrifugation, the supernatants containing the recombinant HA-tagged VHHs were used to immunostain HT1080 cells. Briefly, 50 µl of supernatant was mixed with an equal volume of phosphate-buffered serum (PBS) containing 1% bovine serum albumin (BSA), 0.1% Triton X-100 and 100 ng/µl of anti-HA monoclonal antibody. Anti-HA antibody was then revealed with an anti-mouse-IgG antibody conjugated to Alexa Fluor 488, and DNA was stained with 4′,6′-diamidino-2-phenylindole (DAPI). Each well of the plate was imaged with the Arrayscan high-content screening reader (Cellomics) using a 20× objective.

In all experiments, as negative control, we used a non-specific sdAb that appeared negative in the immunostaining screening and in immunoassays, despite being well expressed and purified from bacteria. Without knowing antigen specificity, we refer to it as control VHH.

### Plasmids

The pHEN4 vector was modified to express the recombinant VHH with a HA–His6 C-terminal tag in *E. coli*. For this, the NotI–EcoRI fragment was replaced with a sequence encoding the HA–His6 fusion, by oligonucleotide hybridization and ligation into the NotI/EcoRI digested pHEN4-VHH vector (primers 1 and 2, Table S1). To express the VHH C-terminally fused to GFP, the VHH–HA sequence was amplified from the pHEN4 vector (primers 3 and 4, Table S1), digested with XhoI and BamHI and cloned into the pEGFP-N1 vector (Clontech). For lentiviral transduction, the NheI-BamHI fragment of the pEGFP-N1, containing the VHH–HA sequence, was sub-cloned into the pTRIP-GFP vector (gift from Louis Van Den Berghe, Toulouse, France). For insertion in the *Drosophila* genome, the XhoI-XbaI VHH–HA–GFP sequence from pEGFP-N1 was subcloned into the pUAS AttB vector (Bischof et al., 2007).

GFP-tagged bivalent chromatibody constructs were established by inserting a linker to the HindIII-PstI sites of the pEGFPN1 chromatibody construct, upstream from the chromatibody sequence. This linker, obtained by hybridizing two oligonucleotides (primers 5 and 6, Table S1), introduced the hinge coding sequence of the llama γ2c (Conrath et al., 2001). The resulting plasmid was used to clone the chromatibody sequence (PCR using primers 3 and 7, Table S1) to the XhoI-HindIII sites (i.e. upstream from the hinge sequence) to generate the chromatibody–chromatibody–GFP fusion. To generate the chromatibody–GFP–chromatibody construct, the chromatibody–HA sequence was cloned into the XhoI-BamHI sites of the pEGFP-C1 vector (Clontech), using primers 8 and 9 (Table S1), resulting in the pEGFP-C1 chromatibody plasmid. Then, the NheI-BsrG1 fragment of the pEGFP-N1 chromatibody, encoding the chromatibody–GFP, was cloned into the pEGFP-C1 to generate the chromatibody–GFP–chromatibody bivalent construct.

### VHH purification and labeling

Chromatibody or control VHH were produced in the BL21 DE3 *E. coli* strain (New England Biolabs). 2xTY medium, supplemented with 100 µg/ml ampicillin and 0.1% glucose, was inoculated with a fresh colony, and the culture was grown at 37°C under agitation (220 rpm). When the optical density at 600 nm (OD_600_) reached 0.7, induction was performed (0.5 mM IPTG) at 28°C for 16 h. The VHHs were purified from periplasmic extract or directly from the culture supernatant in a two-step process involving an ammonium sulfate precipitation (65% saturation) followed by an immobilized metal affinity chromatography (cobalt resin, Thermo Scientific). After imidazole elution, the buffer was exchanged to PBS by dialysis, and the concentration of the purified VHH determined by Nanodrop (Thermo Scientific).

### Histone extraction and purification

Histones were purified from HT1080 cells by acid extraction and separated by reverse phase high-pressure liquid chromatography (HPLC) (Shechter et al., 2007). Alternatively, purified recombinant human core histones, the H2A–H2B dimer, the H3.1–H4 tetramer (New England Biolabs) and mononucleosomes (EpiCypher) were purchased.

### Direct enzyme-linked immunosorbent assay

Serial dilution of purified recombinant histones (H2A, H2B, H3.1 or H4), the H2A–H2B dimer, the H3.1–H4 tetramer or mononucleosomes were adsorbed overnight at 4°C onto a microplate (Maxisorp Nunc), blocked for 3 h in PBS 10% skimmed milk and washed in 10 mM Tris-HCl pH 8, 150 mM NaCl and 0.1% Tween 20 (TBST). Primary staining was performed with a mixture of purified HA-tagged VHH (200 ng/ml) with an anti-HA monoclonal antibody (1 µg/ml) diluted in TBST 5% skimmed milk for 1 h. After three washes with TBST, horseradish peroxidase (HRP)-conjugated secondary anti-mouse-IgG antibody (diluted 1:5000 in TBST 5% skimmed milk) was added for 45 min, washed before HRP was revealed with 50 µl of 3,3′,5,5′-tetramethylbenzidine chromogenic substrate (Sigma-Aldrich) added to each well. The reaction was stopped by adding 50 µl of 0.5 M H_2_SO_4_, and the OD_450_ was measured using a microplate reader (Multiskan). Three independent experiments were performed (*n*=3) and each point was performed in triplicate. Mean and standard deviation values were calculated using Microsoft Excel software.

### Molecular modeling

Modeling was performed using the Accelrys Discovery Studio v. 3.1 (DS 3.1). The X-ray structure of the llama anti-cholera toxin VHH domain (PDB code: 4IDL) was identified as the best structural template to homology-model the chromatibody structure. Secondary-structure-guided sequence alignment between the structural template and chromatibody was carried out using the Align 123 program and served as an input for the automated homology modeling program Modeler v. 9.8. We built 50 models and selected the one with the lowest probability density function (PDF) total energy and the best set of DOPE and Profiles-3D scores, reflecting optimal loop refinement and folding consistency, respectively. The X-ray structure of a Kaposi's sarcoma herpesvirus LANA peptide bound to the nucleosome core (PDB code: 1ZLA) provided the H2A–H2B dimer coordinates and a framework to model the interaction with chromatibody. The chromatibody CDR3 hairpin structure was prepositioned into the H2A–H2B acidic cavity by superimposing its CBM-like motif backbone (RLLSTGR, residues R105–R111) onto that of LANA CBM (residues K6–K12). The resulting complex structure was further processed and minimized using the Charmm27 force field. The same protocol was applied to dock the chromatibody CDR3 hairpin into the H2A–H2B acidic cavity at the surface of the nucleosome core. The criteria used for identifying hydrogen bonds or hydrophobic interactions were a donor–acceptor distance of ≤2.5 Å or a distance ≤5 Å, respectively.

### Cell culture, transfection and transduction

HT1080, HCT116, HeLa and HEK 293T cell lines (ATCC) were grown in DMEM Glutamax^®^ with 4.5 g/l glucose (Life Technologies), supplemented with 10% fetal bovine serum and antibiotics when necessary (penicillin-streptomycin, Life Technologies), and were regularly tested for contamination. Transient transfections were performed with JetPEI reagent (PolyPlus) according to the manufacturer's instructions. Lentiviral particles were produced by co-transfecting HEK 293T cells with the pTRIP vector (containing the gene of interest), the Gag-Pol 8.91 and the VSV-G plasmids. The cell medium containing the generated lentiviruses was used to transduce HT1080 and HCT116 cells.

### Immunofluorescence staining

For immunofluorescence analyses with chromatibody, control VHH or with the anti-53BP1 (1:3000, NB100-304, Novus Biologicals), anti-BRCA1 (1:1000, sc-6954, Santa Cruz Biotechnology), anti-γH2AX (1:3000, JBW301, Millipore) antibodies, cells grown on glass cover slips were fixed using a solution of 4% paraformaldehyde in PBS, permeabilized in PBS containing 0.5% (v/v) Triton X-100, and immunostained using BSA as blocking reagent. For immunostaining with chromatibody or control VHH (HA-tagged), the purified VHH was mixed with a mouse monoclonal anti-HA antibody (HA.11 clone 16B12, Covance) with a 1:5 weight ratio (0.2 µg/ml VHH and 1 µg/ml anti-HA antibody), and the mixture was used for the primary staining. The primary antibodies were revealed with a secondary Alexa-Fluor-488-conjugated anti-mouse- or anti-rabbit-IgG (Life Technologies). The mounting medium was from Dako.

For immunofluorescence analyses with the anti-53BP1, anti-BRCA1 and anti-γH2AX antibodies, cells were scored positive when more than 10 foci per nuclei were detected. Immunostaining with the anti-ubiquitylated H2A (1:100, E6C5, Millipore) and anti-FK2 (1:100, PW8810, Enzo Life Science) antibodies were performed as previously described (Luijsterburg et al., 2012). Immunostained human cells were imaged through a 63× oil objective (NA 1.4) using a Leica DM5000 microscope controlled with MetaMorph acquisition software (Molecular Devices) and equipped with a CoolSNAP EZ camera (Photometrics).

*Drosophila melanogaster* embryos were dechorionated and fixed (Harlow and Lane, 2006). Rehydrated embryos were blocked (PBS with 0.3% Triton X-100 and 1% BSA) and incubated overnight at 4°C in the primary antibody mixture diluted in the same buffer. After extensive washing, the secondary antibody was applied for 5 h and DNA was stained with DAPI. *Caenorhabditis elegans* larvae were fixed with the whole-mount freeze-cracking method and immunofluorescence staining was performed as described previously (Crittenden and Kimble, 2009). Immunostained *Drosophila* embryos and *C. elegans* larvae were imaged with the Leica DM500 wide-field microscope described above, using a 20× (NA 0.7) as well as a 40× (NA 0.85) objective. The protocol used for indirect immunofluorescence labeling of *Saccharomyces cerevisiae* is as described previously (Silver, 2009) and images acquired with a wide-field microscope Leica DM5000 (described above) equipped with a 63× oil objective (NA 1.4).

### *Drosophila* genetics and transgenesis

Wild-type Oregon R *Drosophila melanogaster* was used for immunostaining. For transgenesis, germinal cells of nosC31NLS;attP2 embryos were microinjected with the pUAS AttB:Cb-GFP plasmid and transgenesis was achieved by recombinase-mediated genomic insertion of the AttB UAS chromatibody–GFP expression cassette at AttP sites, at position 68A4 on the third chromosome (Bischof et al., 2007). Ubiquitous zygotic and maternal expression of the chromatibody–GFP transgene was obtained after genetic crossing with fly strains expressing Gal4 under the actin or maternal tubulin promoter, respectively (y^1^ w*; P{w[+mC]=Act5C-GAL4}25FO1/CyO, y^+^, Bloomington stock #4414; or w*; P{matα4-GAL-VP16}V37, Bloomington stock #7073).

### Time-lapse fluorescence microscopy

HT1080 or HCT116 cells stably expressing the chromatibody–GFP fusion were grown in calf-serum-complemented DMEM medium in chambered coverglass (Lab-Tek Nunc). Time-lapse fluorescence microscopy was performed using an inverted wild-field microscope (Zeiss Axio Observer Z1) controlled with the MetaMorph software (Molecular Devices), associated with a CoolSNAP ES2 camera (Photometrics), and equipped with an incubation chamber (Pecon) with controlled temperature (37°C), humidity, and CO_2_ (5%) parameters. The acquisitions were performed using a 20× NA 0.5 or a 40× NA 0.95 objective at 5-min intervals. *Drosophila* embryos were collected on 3% agar plates supplemented with grape juice, manually dechorionated, and placed in a drop of halocarbon oil deposited in a 35-mm cell culture dish with a glass bottom (Greiner Bio-one) prior to live imaging. Time-lapse fluorescence imaging of embryos was performed using a 25× objective (NA 0.8) of a laser-scanning microscope (LSM 510 Zeiss) in inverted configuration, equipped with a controlled temperature (25°C) and moisture incubation chamber (Pecon). The image acquisition frequency was 2 min. Live larvae and adults expressing the fluorescent chromatibody were imaged by epi-fluorescence illumination with a MacroFluo (Leica) equipped with a CoolSNAP ES2 camera (Photometrics). All the imaging data were processed using Fiji (ImageJ NIH) software.

### FRAP analysis

Fluorescence recovery after photobleaching (FRAP) experiments were performed using a confocal laser-scanning microscope (Zeiss LSM510) in inverted configuration, equipped with a 40× (NA 1.2) immersion objective and an incubation chamber, controlled by Zen software (Zeiss). Photobleaching and GFP excitation were performed with the 488 nm line of an argon laser. A small (1.2 µm) circular region of interest (ROI) was arbitrarily positioned in the nucleus. The GFP bleaching acquisition sequence was a pre-bleaching acquisition, bleaching of the ROI (single scan 163.8 µs pixel dwell at 100% laser power), followed by an acquisition every second. The FRAP module of the Zen software (Zeiss) was used to determine the mean ROI intensity value, the relative mean ROI for each time point, and to fit the FRAP recovery curves to the mono-exponential function I(*t*)=I0−I1.e(−*t*/T1). In this equation, I0 is the end value of recovered fluorescence intensity, I1 is the amplitude of the recovered fraction, and T1 is related to the recovery half-time (*t*_1/2_) as determined by *t*_1/2_=−T1.ln(0.5). Each curve was obtained by plotting the mean relative intensities, calculated from nine independent FRAP time courses, as a function of time after bleaching (error bars are s.d.).

### Protein electrophoresis and immunoblotting

Whole-cell extracts were prepared by direct lysis of the cells in SDS loading buffer. Proteins were separated by denaturant electrophoresis. For immunoblotting, proteins were transferred to nitrocellulose. After blocking, the blots were incubated in TBST 5% skimmed milk containing purified VHH–HA (200 ng/ml) and an anti-HA mouse monoclonal antibody (1 µg/ml, HA.11 clone 16B12, Covance), anti-H2A rabbit polyclonal (1:1000, Ab15653, Abcam), anti-H2B rabbit monoclonal (1:1000, D2H6, Cell Signaling) or anti-ubiquitylated H2A mouse antibody (1:500, E6C5, Millipore). The primary antibodies were detected with HRP-conjugated anti-mouse- or anti-rabbit-IgG antibodies and enhanced chemiluminescence (West Dura Thermo Scientific), and photon emission recorded with a camera (G-box Syngene).

### Cell fractionation

Cell fractionation was carried out as previously described (Bonisch et al., 2012). HT1080 cells stably expressing either chromatibody–GFP or H2B–GFP were grown on 100-mm dishes, trypsinized, washed twice with PBS, and washed in ice-cold hypotonic buffer (10 mM HEPES pH 8, 10 mM KCl, 1.5 mM MgCl_2_, 0.34 M sucrose, 1 mM DTT and complete protease inhibitors). Cells were pelleted, resuspended in chilled hypotonic buffer containing 0.2% NP40 and incubated for 10 min on ice. After centrifugation (6500 ***g*** for 5 min at 4°C), the supernatant was kept as the soluble cell fraction and the pellet was washed in 1 ml of cold hypotonic buffer, before the nuclei were lysed in 1 ml of SDS-PAGE loading buffer and kept as the chromatin fraction. The fractions were analyzed by western blotting, with antibodies directed against GFP or H2B.

### Statistical analyses

For ELISA assays, the results were analyzed using the GraphPad Prism 6 software for Windows. The statistical differences were analyzed by two-way ANOVA (multiple comparisons) and are indicated by dollar symbols in the figures. When ANOVA showed a statistical difference (*P*<0.05), comparison among data was performed using Tukey's multiple comparisons test or Bonferroni's multiple comparisons test (between chromatibody and control VHH). For DNA damage response (DDR) alteration, statistical differences were evaluated using a Student's *t*-test in Microsoft Excel and are indicated by asterisks in the figures. *P*<0.05, *P*<0.01, *P*<0.001 or *P*<0.0001, indicating a statistical significance, are noted with dollars or asterisks ($ or *, $$ or **, $$$ or *** and $$$$ or ****, respectively).
